# Catastrophic shear-removal of subcontinental lithospheric mantle beneath the Colorado Plateau by the subducted Farallon slab

**DOI:** 10.1038/s41598-019-44628-y

**Published:** 2019-05-31

**Authors:** David Hernández-Uribe, Richard M. Palin

**Affiliations:** Department of Geology and Geological Engineering, Colorado School of Mines, 1500 Illinois St, Golden, CO 80401 USA

**Keywords:** Tectonics, Geodynamics, Petrology

## Abstract

The causes of Cenozoic uplift of the Colorado Plateau, southwestern USA, are strongly debated, though most hypotheses acknowledge the importance of northwest-directed subduction of the Farallon oceanic plate beneath North America since c. 100 Ma. Existing thermomechanical models suggest that the Farallon slab underthrust the proto-plateau region at ~200 km depth, removing the basal portions of its subcontinental lithospheric mantle (SCLM) root, although such small-volume subduction erosion cannot fully account for the degree of uplift observed today. Here we show via petrological modeling of lawsonite-bearing eclogite xenoliths exposed in diatremes in the center of the plateau that the Farallon slab surface penetrated through the proto-plateau SCLM at much shallower depths (~120 km) than these previous estimates, allowing shear-removal of ~80 km of SCLM – a volume up to three-times greater than previously suggested. This removal led to asthenospheric upwelling and isostatic rebound of the plateau region during the late Cretaceous to the Eocene. We posit that similar shear-removal of SCLM likely played a major role in inhibiting cratonic growth and stabilization in the Neoarchean and Paleoproterozoic – when low-angle subduction of oceanic lithosphere was more prevalent than today – accounting for the atypically thin roots existing below many ancient cratons worldwide.

## Introduction

**Figure 1:**
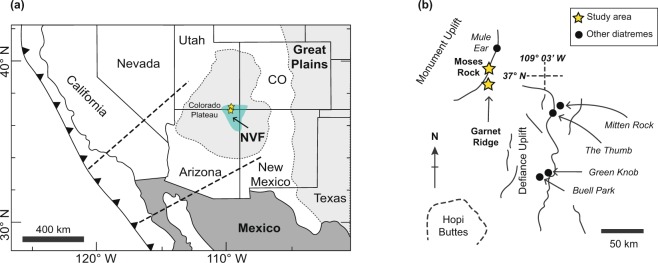
(**a**) Location of the Colorado Plateau and the Navajo Volcanic Field (NVF) in the central part of the plateau. The dashed lines from the trench indicates the approximate direction of Farallon–North America plate convergence and the approximate limits from the flat slab^28^. (**b**) Schematic map showing the different diatremes within the NVF. Yellow stars mark the location of the Garnet Ridge (xenolith 17GR11) and the Moses Rock diatremes (xenoliths 17MSR9 and 17MSR11). Modified from Usui *et al*.^34^.

The present-day elevation, thickness, and lithospheric structure of the Colorado Plateau, southwestern USA, is well-constrained by satellite observations^1^, digital elevation models^2^, and regional-scale geophysical investigations^3–8^. Widespread surface uplift and plateau formation at c. 25 Ma relative to the adjacent, low-elevation Great Plains (Fig. 1a), has been attributed to crustal thickening, thermal expansion, shear-removal or density-driven delamination of mantle lithosphere, or combinations of each^9–14^. Nonetheless, in all cases, the timing and mechanism of plateau formation appear to be intimately related to low-angle underthrusting of the Farallon oceanic slab beneath the southwestern USA since c. 100 Ma^15,16^.

Shallow-angle subduction of oceanic lithosphere (<30° below horizontal) is an uncommon process on Earth today, but is an important mechanism for crustal recycling, as it promotes transport of material from the overriding plate to the subduction channel via subduction erosion^17^. While this material is often eroded proximal to the trench, flat subduction may facilitate subcontinental lithospheric mantle (SCLM) removal from the overriding plate far inland of the plate boundary, affecting its buoyancy^18^. Regional-scale numerical models of Farallon slab subduction over the past ~100 Myr suggest that the angle of dip of the slab has decreased over time^19–21^, although estimations of its paleo-depth below the continental USA at the time of eclogitization and the mass of lithospheric mantle displaced or removed by its progression are wide-ranging^9,20,22^. As such, there remains uncertainty surrounding the degree of subducted slab–SCLM interaction and the extent to which it may have driven uplift of the Colorado Plateau during the late Cretaceous to Eocene^23,24^.

Here, we present new petrological data from lawsonite-bearing eclogite xenoliths sampled from the c. 30–20 Ma Navajo Volcanic Field (NVF), central Colorado Plateau (Fig. 1), which record tectono-metamorphic conditions along the subducted Farallon plate surface prior to their exhumation via diatreme emplacement. Phase diagram-based thermobarometry constrains peak metamorphic eclogite-facies conditions to ~35–37 kbar and ~615–625 °C, equivalent to a slab-top depth of ~120 km, assuming a three-layer rheological model for the proto-Colorado Plateau lithosphere. Geophysical profiles of the adjacent and undeformed Great Plains, eastern Colorado, show SCLM to a depth of ~200 km^25^, indicating large-scale shear-removal of at least ~80 km of the pre-uplifted Colorado Plateau’s mantle keel – notably larger than volumes predicted by previous thermomechanical and petrological models. This effect is likely to have been significantly important in the Late Archean and Proterozoic, when low-angle subduction of oceanic lithosphere was more prevalent than today.

## Geological setting

The Colorado Plateau is a broad region (~337,000 km^2^) of high mean elevation (~2000 m above sea level) located in the southwestern interior of the USA (Fig. 1a). It largely comprises unmetamorphosed and undeformed Paleozoic to Cenozoic sedimentary rocks overlying high-grade metamorphic Precambrian basement^26,27^. It is bordered by the Basin and Range province to the northwest, by the Rocky Mountains to the northeast, and to the southeast by the Rio Grande Rift, all of which experienced significant Cenozoic orogenic activity and extensional tectonics^14,27^. Tectonic reconstructions of the recent geological history of the Colorado Plateau indicate various stages of uplift related to the northeastward subduction of the Farallon oceanic plate beneath the southwestern USA during the Late Cretaceous and Early Cenozoic^23,24,28^, although the exact geodynamic processes responsible remain subject to debate^29^.

Cenozoic volcanic edifices within the NVF, central Colorado Plateau (Fig. 1), comprise minettes and serpentinized ultramafic microbreccias^30^. These diatremes contain a wide variety of crustal and mantle xenoliths that document the petrological constitution of the entire continental lithosphere prior to their exhumation. The origin of rare lawsonite-bearing eclogite xenoliths within these diatremes is strongly debated: some studies interpret them to represent exhumed fragments of the subducted Farallon oceanic plate^31^, whereas other studies suggest that they represent metamorphosed fragments of the overriding lower continental crust that were scraped off its base close to the trench due to subduction erosion, and subsequently pushed northeastwards by the leading edge of the Farallon plate^32^, where they were later exhumed during diatreme formation. It is notable that while these contrasting origins are not mutually exclusive – and both ‘types’ likely occur – the metamorphic evolution experienced by these mafic protoliths would have been almost identical, as each would be expected to record the changing pressure–temperature (*P*–*T*) conditions experienced by the top of the Farallon slab. Zircon and monazite from eclogitic xenoliths have produced U–Pb ages of c. 80–30 Ma^31–33^, which are interpreted to date prograde-to-peak metamorphism and progressive devolatilization during subduction.

Due to variable degrees of retrogression, conventional thermobarometry has so-far been unable to place precise constraints on the metamorphic *P*–*T* history of eclogite xenoliths from this region. Temperatures of peak metamorphism are fairly well constrained at ~560–700 °C, although corresponding pressure estimates made by previous workers range from ~26 kbar to ~50 kbar^31–33^, implying that the top of the Farallon slab was located somewhere between ~100–200 km depth. While this lower bound is supported by coesite inclusions in garnet^31^, which requires ultrahigh-pressure (UHP) conditions (>27 kbar) to stabilize, this upper-limit uncertainty significantly hinders reliable geometric reconstruction and/or validation of geodynamic models of slab subduction beneath the proto-plateau during the Cenozoic.

### Petrology of eclogite xenoliths

During fieldwork conducted in June 2017, multiple samples of mafic eclogite xenoliths were collected from various diatremes in the NVF (Fig. 1b). In this work, we present data from three samples from Garnet Ridge (17GR11) and Moses Rock (17MSR09 and 17MSR11), which best preserve peak metamorphic assemblages and show the least petrographic evidence for retrogression. These xenoliths are interpreted to represent exhumed portions of the uppermost surface of the oceanic Farallon slab, as opposed to lower-crustal materials removed via subduction erosion, based on trace-element geochemical ratios distinguishing a MORB source for all three samples (Supplementary Fig. 1). These data agree with the interpretations of other workers who studied similar lawsonite-bearing eclogites from diatremes in the northwestern corner of the NVF, also interpreting them to be fragments of subducted oceanic crust^34^. Mineral proportions and a detailed mineral characterization of the individual samples are provided in Supplementary Figs 2–4, and representative mineral compositions are shown in Supplementary Table 1.

**Figure 2:**
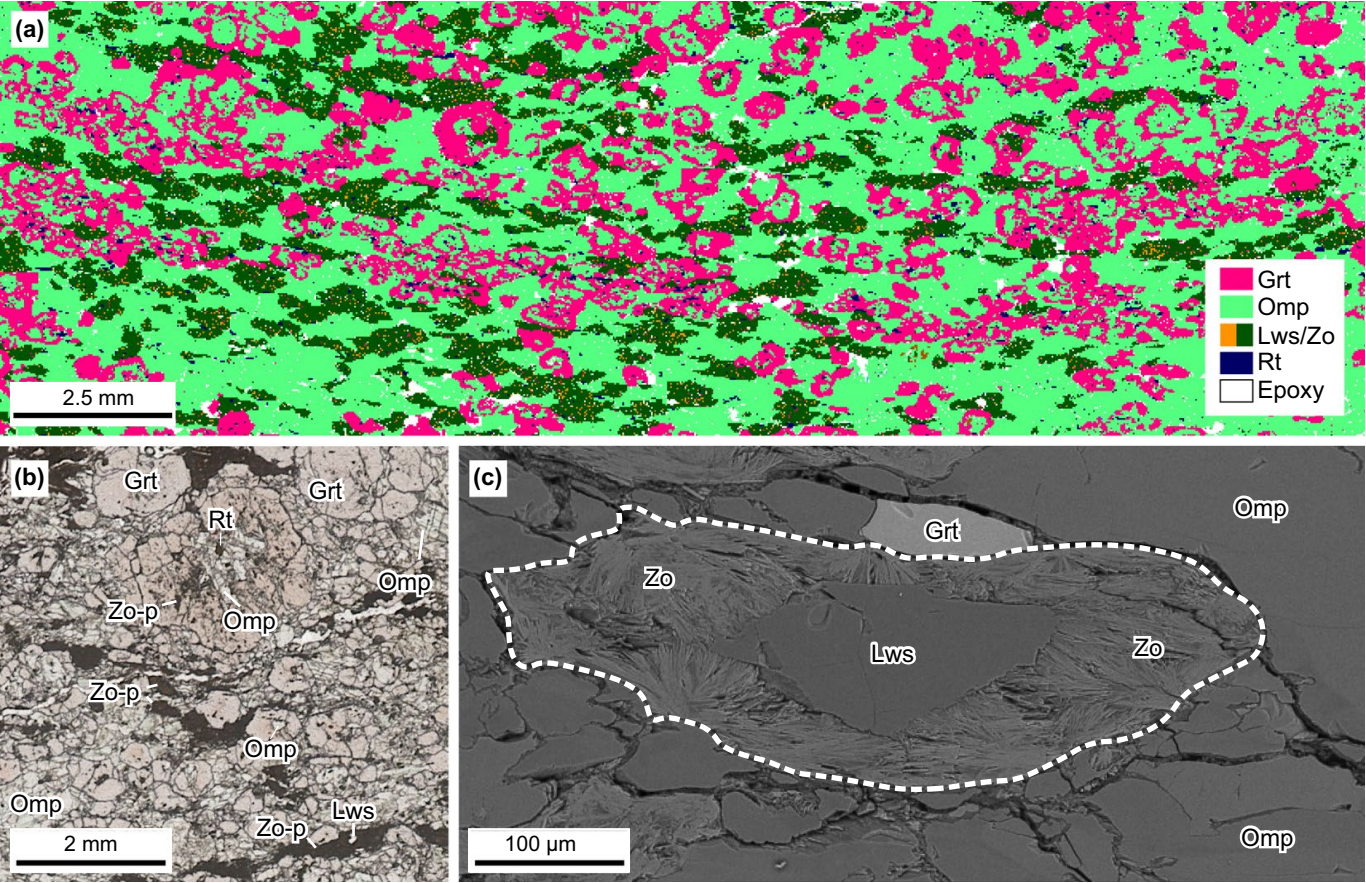
Representative mineralogical and textural features from NVF eclogite xenoliths. (**a**) Automated mineralogy scan of 17GR11 showing detailed mineral identification (see Methods). (**b**) Plane-polarized light photomicrograph showing textural relations between eclogite-facies mineral assemblages. (**c**) Back-scattered electron image showing partial pseudomorphic replacement of lawsonite by acicular zoisite. Original crystal boundary is marked by a dashed line. See Methods for mineral abbreviations. Zo-p = Zoisite pseudomorph after lawsonite.

**Figure 3:**
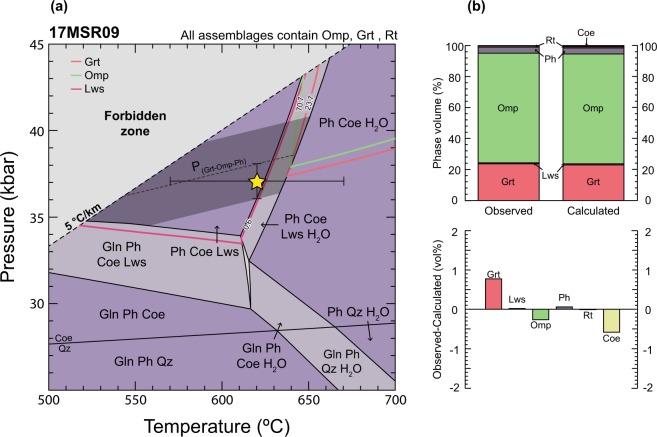
Petrological model for eclogite 17MSR09. (**a**) Pressure–temperature phase equilibrium diagram. The yellow star indicates the peak *P*–*T* conditions that provide the best match between observed and calculated mineral proportions. Error bars are ±1 kbar and ±50 °C at the 2σ level^38,39^. The grey shaded area represents constraints from the garnet–omphacite–phengite barometry, with calculated conditions lying along the dashed line (P_Grt-Omp-Ph_) and an uncertainty envelope of ±2 kbar. Solid lines represent calculated mineral volume proportions matching observations. (**b**) Comparison between the observed and calculated mineral proportions at ~37 kbar and ~620 °C, represented by the yellow star in (**a**). See methods for mineral abbreviations and details on the modeling.

**Figure 4:**
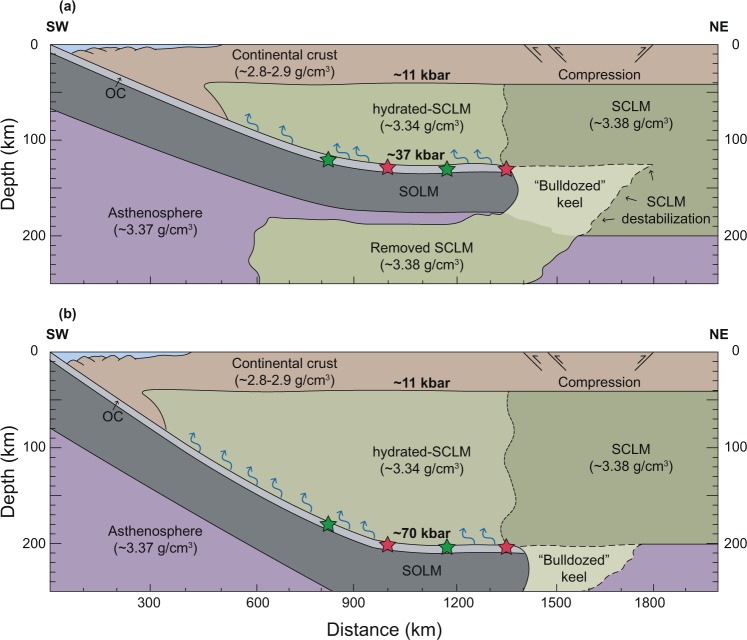
Schematic cross-section of Farallon flat subduction during the Laramide orogeny. (**a**) Tectonic model in accordance to our *P*–*T* results and calculations. (**b**) Tectonic model showing the pressure necessary to account for the dip-angle and depths of the Farallon plate predicted in Liu and Currie^19^ and Axen *et al*.^20^ 2-D geodynamic models. Location of deformation according to Axen *et al*.^20^. Green stars represent eclogites with a protolith derived from the Farallon plate. Red stars represent eclogites with a protolith scraped-off from the overriding plate. OC = Oceanic crust; SOLM = Sub-oceanic lithospheric mantle; SCLM = Subcontinental lithospheric mantle. Density estimates correspond to Hacker *et al*.^45^, Lee *et al*.^46^, Griffin *et al*.^57^, and Dziewonski and Anderson^61^, for continental crust, hydrated-SCLM, SCLM, and asthenosphere, respectively.

All xenoliths contain garnet (~19–30%), omphacite (~57–80%), lawsonite (~1%), zoisite (~1–15%), and rutile (~1%) (Fig. 2a). Sample 17GR11 contains additional quartz/coesite^31^ (~1%) and 17MSR09 has additional phengite (4%). Microtextural relationships suggest that the peak eclogite-facies mineral assemblage comprised garnet + omphacite + lawsonite + rutile ± phengite ± coesite^31^. Garnet occurs as small- to medium-sized porphyroblasts up to ~1 cm in diameter (Fig. 2a,b) that preserve compositional zoning from core (Alm_69–64_, Grs_29–22_, Prp_10–7_, Sps_9–3_) to rim (Alm_55–43_, Prp_45–25_, Grs_15–9_, Sps_2–1_) (Supplementary Table 1 and Figs 2–4). Core domains contain inclusions of omphacite, rutile, quartz/coesite^31^, lawsonite, and zoisite pseudomorphs after lawsonite (Fig. 2b), whereas rims occasionally contain inclusions of omphacite, rutile, and quartz/coesite^31^ (Fig. 2a,b). Some porphyroblasts show atoll textures with cores completely replaced by omphacite (Fig. 2a,b). Lawsonite in all samples shows variable degrees of pseudomorphic replacement by fine-crystalline, acicular zoisite, which show inward-directed spray-like morphologies (Fig. 2c). This microtexture suggests lawsonite replacement during exhumation^31^, as the original euhedral crystal boundaries of the grains are well preserved (Fig. 2c). Omphacite in the matrix (Na/(Na + Ca) = 0.47–0.65; Supplementary Table 1) shows equilibrium textural relationships with garnet, matrix lawsonite and zoisite pseudomorphs (Fig. 2a), indicating equilibrium co-existence at peak conditions. Phengite in 17MSR09 has a Si content of 3.61–3.72 cations per formula unit (Supplementary Table 1), supporting the interpretation that it was stable at UHP conditions^35^.

## Thermobarometry and Geodynamic Interpretation

### Petrological modeling

Peak *P*–*T* conditions for all eclogite xenoliths prior to exhumation were calculated via petrological phase equilibrium modeling, which employs an iterative Gibbs free energy minimization procedure to determine the most stable mineral, fluid, and/or melt assemblage that would form at specific *P*–*T* conditions in a fixed bulk-rock composition^36,37^. Peak *P*–*T* conditions at which each xenolith likely equilibrated were obtained by correlating predicted mineral proportions with the values measured in each sample (Fig. 3 and Supplementary Figs 5–7), whilst considering all associated uncertainties^38–40^. Model set-up parameters and activity–composition (*a*–*x*) relations considered for phases exhibiting solid solutions are described in the Methods section.

A representative *P*–*T* phase equilibrium diagram for xenolith 17MSR09 is shown in Fig. 3a. The observed peak assemblage Grt–Omp–Lws–Rt–Coe–Ph is stable at *P* > 35 kbar and *T* < 640 °C. This field is limited at lower pressure by the stabilization of glaucophane – which is not observed within the stable assemblage – and at higher temperatures by the consumption of lawsonite. Isolines of equal volume proportion of garnet, omphacite, and lawsonite constrain peak metamorphism at ~37 kbar and ~620 °C, with associated 1σ uncertainties of ~1 kbar and ~50 °C^38,39^. This pressure for the Farallon slab surface thus infers a minimum depth of equilibration of ~120 km, assuming lithostatic conditions, as outlined below. Petrological modeling results for xenoliths 17GR11 and 17MSR11 are shown in Supplementary Figs 6 and 7 and record equivalent *P*–*T* conditions for peak metamorphism within error. An independent estimate of peak metamorphic pressure for sample 17MSR09 was obtained via the garnet–omphacite–phengite barometer calibration for white mica-bearing eclogites^41^. Compositions for garnet rim domains and adjacent matrix omphacite and phengite in textural equilibrium (Supplementary Table S1) produced a pressure of ~38 kbar at 620 °C, with an uncertainty of ±2 kbar (Fig. 3a). This equilibrium has a positive slope in *P*–*T* space with d*T*/d*P* = 40 °C/kbar and passes through the center of the interpreted peak assemblage field, as constrained by the observed phase assemblage and mineral proportions.

We limit the calculated peak conditions of metamorphism to lie outside of the ‘forbidden zone’, defined by (Liou, Hacker & Zhang 2000)^42^ as geothermal gradients colder than 5 °C/km. This geotherm represents the minimum rate of conductive heating that rocks may experience during descent into the Earth, and no known entirely mafic crust is documented to have experienced such *P*–*T* conditions. While some studies report metamorphic conditions that lie within this high-pressure/low-temperature domain (e.g. Zhang *et al*.^43^), these occurrences are exclusively UHP terranes formed via deep subduction of continental crust, which provides positive buoyancy for rapid exhumation and preservation. Subducted oceanic crust or eroded mafic fragments from the base of an overlying arc subjected to thermal diffusion over periods longer than ~5–10 Myr are expected to thermally equilibrate, as the characteristic diffusion distance for this time scale at mantle conditions is ~10–15 km^41^. Thus, it is unlikely that the uppermost or middle portions of the Farallon slab crust could preserve such low thermal gradients, as geochronology of eclogite xenoliths indicates that the experienced prograde metamorphism for at least 45 Myr^33^.

### Dip-angle of the Farallon plate and thickness of the SCLM

Existing thermomechanical models of Farallon slab evolution over time suggest that flattening was promoted by the subduction of an oceanic plateau at c. 90 Ma^44^. The c. 80–30 Ma age range recorded by eclogite xenoliths^31–33^ from the NVF suggests that metamorphic recrystallization was prevalent during the low-angle and flattened subduction stages of the Farallon plate. Thus, we interpret the peak *P*–*T* conditions determined from xenoliths 17MSR09, 17GR11, and 17MSR11 in this work (Fig. 3 and Supplementary Figs 6 and 7) to represent the maximum depths reached by the top of the plate during these stages, assuming that it remained at approximately the same depth once it flattened and migrated inboard of the continent.

The results of thermobarometry shown here constrain the depth and dip-angle of the Farallon plate during this period, and so the thickness of the SCLM below the Colorado Plateau (see Supplementary Methods). In a two-layer model that considers representative densities for the upper and lower continental crust^45^, the continental Moho beneath the proto-plateau would have been at a pressure of ~11 kbar (Fig. 4a). The pressure difference of ~26 kbar between this value and that calculated here for the Farallon plate slab surface (i.e. ~37 kbar from eclogite 17MSR09; Figs 3a and 4a), combined with a representative mantle density of 3.34 g/cm^3^ from mantle xenoliths recovered from the SCLM beneath the Colorado Plateau^46^, implies an intermediate SCLM root ~80 km in thickness (Fig. 4a). The Farallon slab-top was therefore located at ~120 km depth, in agreement with the present lithospheric thickness of the Colorado Plateau^7,25^ (Fig. 4a). As the Colorado Plateau and adjacent Great Plains (Fig. 1a) share similar Mesozoic geological histories and overlie the same Proterozoic terranes^47^, geophysical measurements of the thickness of SCLM beneath the latter today (~200 km)^25^ imply a similar thickness beneath the former prior to shear removal. Consequently, over ~80 km of SCLM must have been removed from the base of the proto-plateau’s root due to northeastward progression of the subducted Farallon plate, which is greater than estimates provided by recent studies (~20–50 km)^20^.

If this depth is considered the maximum reached by the Farallon plate slab-top prior to and/or during flattening, the mean dip angle of the slab during the Late Cretaceous to the Eocene can also be estimated from our *P*–*T* data. Current geodynamic models suggest that the Farallon slab flattened ~1000 km inboard of the oceanic trench during the early stages of the Laramide orogeny^19–21^ (Fig. 4a). If correct, during low-angle subduction and prior to flattening, the slab would have required a mean dip angle of ~7°. Sensitivity analysis considering geologically realistic uncertainties for these geometric data indicates that the mean dip angle may change by ±0.6° for every 10 km uncertainty in proto-plateau continental crust thickness and ±0.3° for every 50 km variation in absolute distance from the trench (Supplementary Fig. 8). Calculated SCLM thickness may vary by ±3 km for every 1 kbar of pressure variation and ±1 km for every 50 kg/m^3^ change in density (Supplementary Fig. 9), although the absolute magnitude of error associated with pressure determination via petrological phase equilibrium modeling is considered to be less than ~1 kbar for well-equilibrated parageneses^38^.

## Discussion

Constraining the rates and styles of lithospheric-scale geodynamic processes has critical importance for validating our understanding of how the Earth has evolved throughout geological time. Furthermore, such *P*–*T*–*t* data obtained via petrological modeling are routinely used as benchmarks and/or constraints for numerical models of lithospheric evolution. If our knowledge of the rates and styles of metamorphism and tectonic deformation are poorly constrained, the results and implications drawn from thermo-mechanical simulations become less reliable.

While eclogite xenoliths from the NVF have been studied by many workers^31–33^, all previous *P*–*T* estimates for peak conditions have been determined via conventional thermobarometry, which is subject to large inter-calibration uncertainties and relies on measured mineral compositions accurately representing those attained at the metamorphic peak^38,48^. This is exemplified by the wide range of estimated peak pressures reported for eclogite xenoliths (~27–50 kbar)^31–33^, which necessarily must have been exhumed from the same paleo-slab top due to their spatially restricted occurrence in the northwestern NVF. However, over the past decade, petrological phase equilibrium modeling has become the preferred technique with which to perform reliable forward and inverse modeling of subduction zone processes^49,50^ as it employs a multi-equilibrium approach combining internally consistent thermodynamic data and more geologically realistic *a*–*x* relations for minerals with solid solutions. The peak *P*–*T* conditions obtained here using this technique (~35–37 kbar and ~615–625 °C; Fig. 3 and Supplementary Figs 6 and 7) lie at the low-pressure end of the range defined by previous studies^31–33^, posing the question of what these alternative higher-pressure estimates mean. One possibility is that conventional thermobarometers in these studies were applied to minerals that were not in mutual chemical equilibrium at the time of metamorphism. For example, relatively minor retrograde diffusional equilibration of garnet rim compositions with adjacent matrix phases in mafic rocks has been demonstrated to cause true *P*–*T* conditions obtained via garnet–omphacite–phengite thermobarometry to vary by up to ±100 °C and ±4 kbar^51^, although this does not seem to be the case in 17MSR09, as the results of garnet–omphacite–phengite barometry match very well with the results obtained via inverse petrological modeling (Fig. 3a).

An alternative solution to these discrepancies is the influence of tectonic overpressure that complicates the conversion of pressure to depth within the Earth. Recent studies have shown that localized, non-lithostatic overpressure may occur to different degrees through a lithospheric column in convergent tectonic settings^52,53^. Further, relatively rigid rheological components preferentially act as foci for overpressure, such as cold and dense oceanic crust in direct contact with hotter and more malleable mantle lithosphere^54^. Modeled tectonic overpressure at the surface of subducted oceanic crust at ~120 km has been calculated to potentially reach magnitudes of 1–5 kbar^55^, yet this is too small to account for the absolute pressure differences reported between studies in this case. It is critical to note that any component of non-lithostatic overpressure within the total pressure calculated in our modeling procedure would have the effect of decreasing the depth of subduction of the Farallon slab, meaning that it would have penetrated through the proto-plateau’s lithospheric root at even shallower depths and sheared away even larger mass of SCLM. Thus, the ~120 km reported here must represent a maximum depth, and that the calculated ~80 km of SCLM removed during its advance should thus be considered a minimum.

Prior study of mantle xenoliths from the NVF suggest that the hydrated SCLM beneath the Colorado Plateau has a density of ~3.34 g/cm^3^ (Lee *et al*.^46^) and thus is more buoyant than other SCLM beneath North America^56^, which has a density of ~3.38 g/cm^3^ – similar to that forming the roots of many Archean cratons^57^ (Fig. 4a). Most workers suggest that as the Farallon slab subducted northeast beneath the USA, fluid released from prograde devolatilization reactions – mainly driven by the consumption of chlorite, actinolite, talc and/or lawsonite^58^ – promoted hydration of the overlying SCLM, promoting its chemical depletion via metasomatism^59,60^ (Fig. 4a). Importantly, sheared-away SCLM would not have been exposed to this metasomatism, being positioned at depths below the advancing slab top (Fig. 4).

Thermomechanical modeling shows that a weaker (i.e. hydrated) SCLM that is more chemically depleted will be significantly more conducive to penetration by the Farallon slab during flat subduction^20^, which we suggest allowed this large-scale removal. The relatively dry underlying SCLM would then have a very slight negative buoyancy (Δ*ρ* = −0.01 g/cm^3^, assuming an asthenosphere density of ~3.37 g/cm^3^ from Dziewonski and Anderson^61^), and eventually sink into the underlying mantle (Fig. 4a). Our data therefore support the widely acknowledged hypothesis that lithospheric loss beneath the proto-Colorado Plateau during the Late Cretaceous and/or Eocene was driven by the inboard migration of the subducting Farallon slab^60^, leading to the atypically thin Colorado Plateau SCLM observed in geophysical profiles today^25^. These geophysical studies report a current lithospheric thickness between 120 km and 150 km, which directly correlates with our calculated estimate derived via thermobarometry, but conflicts with the results of some thermos-mechanical models, suggesting deep-seated subduction (Fig. 4b). In these cases, calculated metamorphic pressures of around 70 kbar would be expected from thermobarometric analysis, yet this is not the case for the samples investigated herein. Although the timing of removal of this mass of SCLM is uncertain, upper-mantle seismic tomography indicates that the Farallon slab began to break apart and sink in at least two stages at c. 86–60 Ma^62^ and c. 56–42 Ma^23^. This shear-induced erosion and slab breakaway would then allow asthenospheric upwelling, which has been cited by some workers to be a critical factor in accounting for subsequent plateau uplift^16,23,24,62^. The proposed mechanism of lithospheric thinning by lateral shearing beneath the Colorado Plateau has been suggested by other studies^9,22^, and so our findings potentially provide the first direct constraints on this having occurred in North America.

What are the implications of this result for the efficacy of subduction erosion and removal of continental roots throughout Earth history? While the timing of the onset of subduction within the geological record is unresolved^63^, many studies agree that early plate tectonics involved shallow subduction within a hot Neoarchean–Paleoproterozoic mantle (c. 3.0–1.7 Ga)^64^, with occasional slab tearing and breakoff. This period of Earth history represents a convergence of many major geological transitions, including the widespread emergence of continental crust from beneath the oceans and the associated saturation of Earth’s atmosphere with oxygen (the Great Oxygenation Event)^65^. Sub-horizontal subduction of the Farallon oceanic slab shows fundamental parallels with this early-Earth regime and may further account for the occurrence of some atypically thin Archean cratonic roots worldwide. Thermal estimates of continental lithospheric thickness show a consistent increase with age, from ~100 km in the Phanerozoic to ~250 km in the Early Proterozoic^66^. Archean cratonic lithosphere, however, has a bimodal distribution at ~350 km and ~220 km, despite thermal modeling suggesting an equilibrium thickness of >400 km^66^. Mechanisms such as thermo-mechanical erosion by secondary mantle convection^67^, erosion by mantle plumes^68^, delamination due to Rayleigh-Taylor-type gravitational instabilities in the lower lithosphere^69^, and erosion by basal drag^70^ have all been suggested by previous workers, although our results suggest that the hotter ambient Neoarchean mantle may have alternatively promoted lithospheric shear-removal of these keels, akin to that documented here for the Colorado Plateau. As such, similarities between thinned SCLM in Neoarchean and Phanerozoic terranes provides support for these key geodynamic processes having operated similarly throughout much of geological time.

## Methods

### Petrological characterization of eclogite xenoliths

#### Electron microprobe analysis (EPMA)

Major-element compositional analyses of minerals in each xenolith were acquired using a JEOL JXA 8900 electron microprobe housed at the Denver Microbeam Laboratory at the United States Geological Survey (USGS), Colorado, USA. Both natural and synthetic materials were used as standards for calibration, and a ZAF correction routine was applied. Operating conditions comprised an acceleration voltage of 15 keV, a beam current of 20 nA, and a beam diameter of 5 μm for mica and 1 μm for all other minerals.

#### Automated mineralogy

Volume proportions of minerals in each xenolith were acquired via automated mineralogy, using a TESCAN VEGA-3 model LMU VP scanning electron microscope housed at the Department of Geology and Geological Engineering, Colorado School of Mines, USA. An electron beam was rastered across the surface of a thin section of each xenolith sample at a pixel resolution of 25 µm, using an acceleration voltage of 25 keV and beam intensity of 14.5 nA. Four energy-dispersive detectors simultaneously acquired a backscattered electron (BSE) image at each pixel and a chemical composition. Mineral characterization was performed using both BSE and compositional information, which were compared to spectra stored in an internal database. This procedure produced quantitative mineral abundance maps for each sample, with area proportions in thin section assumed to be representative of volume proportions throughout each xenolith. The calculated proportion of zoisite was considered to represent that for lawsonite at peak metamorphic conditions, as the fine-grained acicular zoisite pseudomorphs retain the original rhombohedral outlines of parent lawsonite, indicating direct replacement with no significant volume change during retrogression^31^. Automated mineralogy scans and obtained proportions for each eclogite xenolith are shown in Supplementary Figs 2–4.

### Thermobarometry

#### Petrological modeling

Phase diagram construction was performed using the Gibbs free energy minimization software Theriak-Domino^71,72^ and the internally consistent thermodynamic data set ds62^73^. Eclogite xenoliths 17GR11 and 17MSR11 were modeled in the nine-component Na_2_O–CaO–FeO–MgO–Al_2_O_3_–SiO_2_–H_2_O–TiO_2_–O_2_ (NCFMASHTO) system, whereas petrological modeling of phengite-bearing xenolith 17MSR09 additionally considered K_2_O. The following *a*–*x* relations for solid-solution phases were used: clinopyroxene (diopside–omphacite–jadeite) and clinoamphibole (glaucophane–actinolite–hornblende)^74^; garnet, biotite, muscovite–paragonite, and chlorite^75^; epidote^73^; plagioclase^76^; and ilmenite^77^. Pure phases comprised talc, lawsonite, kyanite, zoisite, quartz, coesite, and rutile. Mineral abbreviations follow the guidelines of Whitney and Evans^78^. Effective bulk compositions for each xenolith were calculated using mineral proportions derived by automated mineralogy and representative EPMA-derived compositions^79^. Adjustments to measured mineral compositions were made using the “ideal analysis” approach of Powel and Holland^38^, where necessary, to reduce misfit between natural and modeled compositional systems. Individual bulk-rock compositions used to perform phase equilibrium modeling are shown in Supplementary Table 2.

Uncertainties related to the absolute positions of assemblage field boundaries on calculated phase diagrams have been shown to be less than ±1 kbar and ±50 °C at the 2σ level^38,39^, with this variation being largely a function of propagated uncertainty on end-member thermodynamic properties within the data set. However, as all phase diagrams were calculated using the same dataset and *a*–*x* relations, similar absolute errors associated with dataset end-members cancel, and calculated phase equilibria are relatively accurate to within ±0.2 kbar and ±10–15 °C^38,39^. The *P–T* conditions of peak metamorphism for each xenolith were determined by comparing mineral proportions calculated by automated mineralogy against those predicted in each petrological model (Fig. 3 and Supplementary Figs 5 and 6).

## Supplementary information

Supplementary material

## Data Availability

All petrological data necessary to reproduce the results described herein are provided in Supplementary Information. Software enabling petrological calculations (Theriak-Domino) can be downloaded at no cost from the following web address: http://www.rocks.uni-kiel.de/theriakd/html/down_en.html.
