# Supplementary material for: Catastrophic shear-removal of subcontinental lithospheric mantle beneath the Colorado Plateau by the subducted Farallon slab
Source: Sci Rep. 2019 May 31;9:8153. doi: 10.1038/s41598-019-44628-y (PMC6544764; doi:10.1038/s41598-019-44628-y)
Supplement: Supplementary file 1 — Supplementary material [file 41598_2019_44628_MOESM1_ESM.docx]

**Supplementary Information for: Catastrophic shear-removal of subcontinental lithospheric mantle beneath the Colorado Plateau by the subducted Farallon slab**

David Hernández-Uribe^*^ and Richard M. Palin

Department of Geology and Geological Engineering, Colorado School of Mines, 1500 Illinois St, Golden, CO 80401, USA

*^*^corresponding author:* [*dav.hernandez.uribe@gmail.com*](mailto:dav.hernandez.uribe@gmail.com)

**CALCULATION OF THE SUBCONTINENTAL LITHOSPHERIC MANTLE (SCLM) THICKNESS AND DIP ANGLE OF SUBDUCTION OF THE FARALLON PLATE**

Interpretation of subcontinental lithospheric mantle (SCLM) thickness consider the lithosphere beneath the proto-plateau to be multi-layered and in isostatic equilibrium. Lithostatic pressure at the base of the continental crust (*P*_bcc_) (i.e. continental Moho) was calculated using the two-layer model of upper and lower continental crust and their representative densities (equation 1), following Hacker *et al.^1^*

$$eq. \left( 1 \right) P_{bcc}=\left( g*\rho_{ucc}*h_{ucc} \right)+(g*\rho_{lcc}*h_{lcc})$$

where *g* = acceleration due to gravity (9.81 m/s^2^), *ρ*_ucc_ = density of the upper continental crust (2800 kg/m^3^), *h*_ucc_ = thickness of the upper continental crust (14,000 m), *ρ*_lcc_ = density of the lower continental crust (2920 kg/m^3^), and *h*_lcc_ = thickness of the lower continental crust (26,000 m). This calculation produced *P*_bcc_ ~11 kbar.

The thickness of SCLM (*h*_SLCM_) above the uppermost surface of the Farallon slab was determined using the difference between the peak metamorphic pressure obtained from petrological modeling of eclogite 17MSR09 and *P*_bcc_, and a representative density of the SCLM (equation 2):

$$eq. \left( 2 \right) h_{SCLM}= \frac{P_{SCLM}}{(g*\rho_{SCLM})}$$

where *P*_SCLM_ = pressure difference between the top and bottom of the SCLM (26 kbar), *g* = acceleration due to gravity (9.81 m/s^2^), and *ρ*_SCLM_ = density of the SCLM = 3340 kg/m^3^ (Lee *et al.,^2^*). This calculation produced *h*_SLCM_ = 80 km. With this thickness and that for proto-plateau continental crust, we calculated a total depth from the surface to the top of the Farallon plate to be ~120 km.

Equation 3 was used to calculate the mean angle dip (°), assuming that it was constant from the trench. This procedure used the calculated depth from the surface to the slab-top of the Farallon plate, and the distance from the trench to where the Farallon slab flattens, i.e. ~1000 km^3–5^:

$$eq. \left( 3 \right) angle=\tan^{-1} (\frac{h_{tot}}{d_{t-f}})$$

where: *h*_tot_ = total height; depth from the surface to the slab-top of the Farallon plate = 120 km, *d*_t–f_ = the distance from the trench to where the Farallon slab flattens = 1000 km. This produced an angle of ~7°.

**SUPPLEMENTARY TABLES**

**SUPPLEMENTARY FIGURES**

**
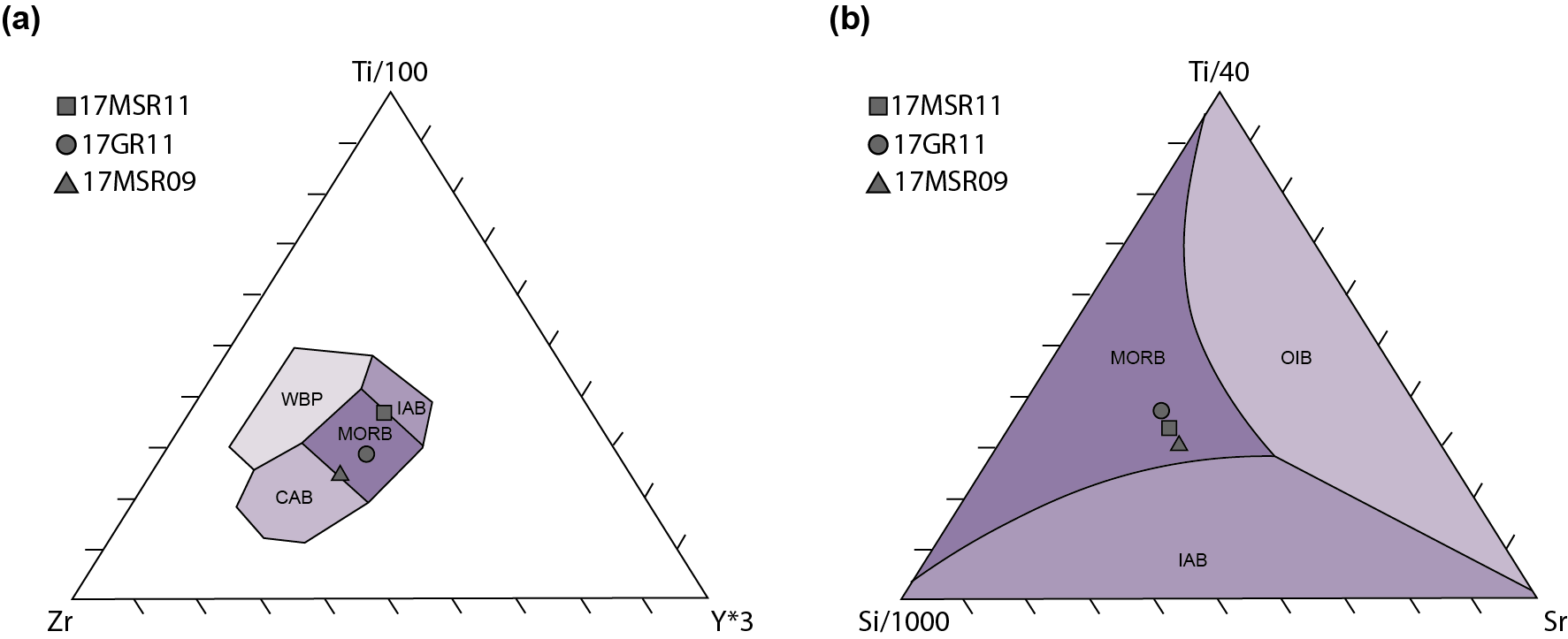
**

**Figure S1.** Tectonic discrimination diagrams for determining the basaltic parentage of lawsonite-bearing eclogites17MSR09, 17MSR11, and 17GR11. (a) Ti–Zr–Y (parts per million; ppm) ternary plot after Pearce and Cann^6^, and (b) Ti–Sr–Si ternary plot after Vermeech^7^. WPB = within-plate basalt; IAB = island-arc basalt; MORB = mid-ocean ridge basalt; CAB = calc-alkaline basalt; OIB = ocean-island basalt.

**
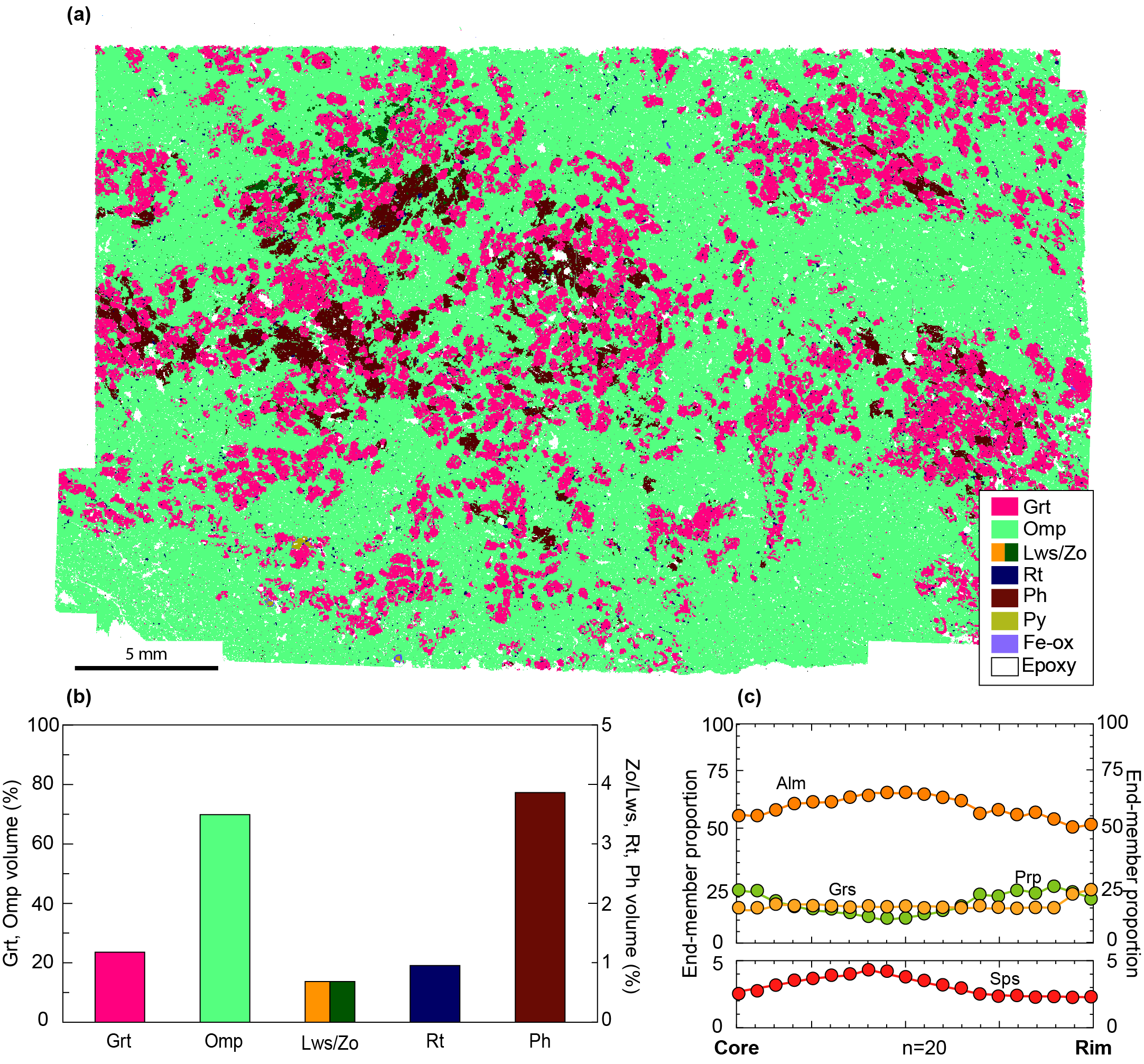
**

**Figure S2.** Petrology of eclogite xenolith 17MSR09. (a) Mineral identification and (b) volume proportions obtained from automated mineralogy. Grt = garnet, Omp = omphacite, Rt = rutile, Ph = phengite, Py = pyrite, Fe-ox = iron-oxides, Zo = zoisite, Lws = lawsonite. (c) Representative garnet analysis showing major element zoning in terms of end-member proportions: Alm = almandine [Fe^2+^/(Fe^2+^+Mg+Ca+Mn)], Prp = pyrope [Mg/(Fe^2+^+Mg+Ca+Mn)], Grs = grossular [Ca/(Fe^2+^+Mg+Ca+Mn)], Sps = spessartine [Mn/(Fe^2+^+Mg+Ca+Mn)].

**
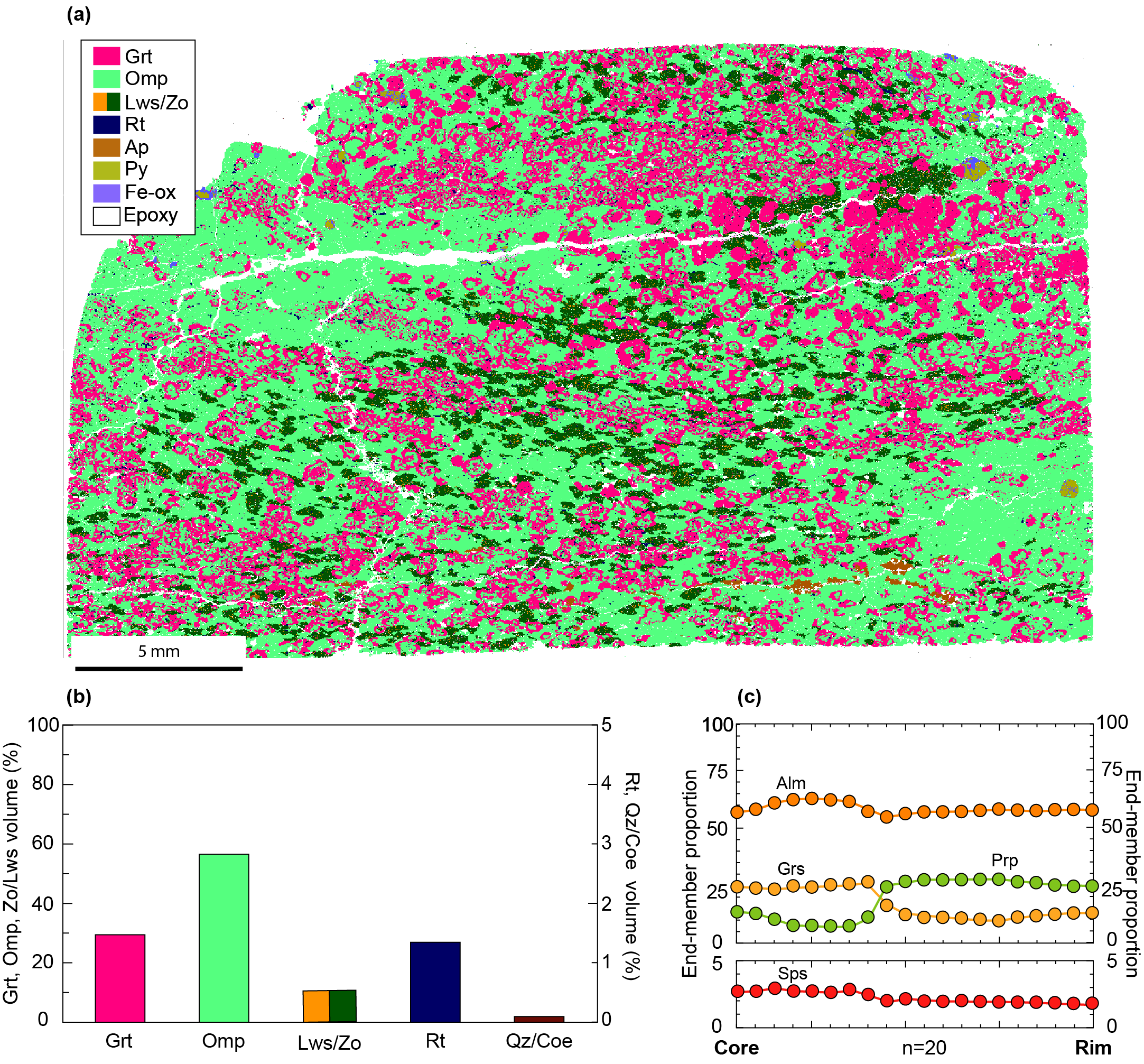
**

**Figure S3.** Petrology of eclogite xenolith 17GR11. (a) Mineral identification and (b) volume proportions obtained from automated mineralogy. Grt = garnet, Omp = omphacite, Rt = rutile, Ap = apatite, Py = pyrite, Fe-ox = iron-oxides, Zo = zoisite, Lws = lawsonite. (c) Representative garnet analysis showing major element zoning in terms of end-member proportions: Alm = almandine [Fe^2+^/(Fe^2+^+Mg+Ca+Mn)], Prp = pyrope [Mg/(Fe^2+^+Mg+Ca+Mn)], Grs = grossular [Ca/(Fe^2+^+Mg+Ca+Mn)], Sps = spessartine [Mn/(Fe^2+^+Mg+Ca+Mn)].

**
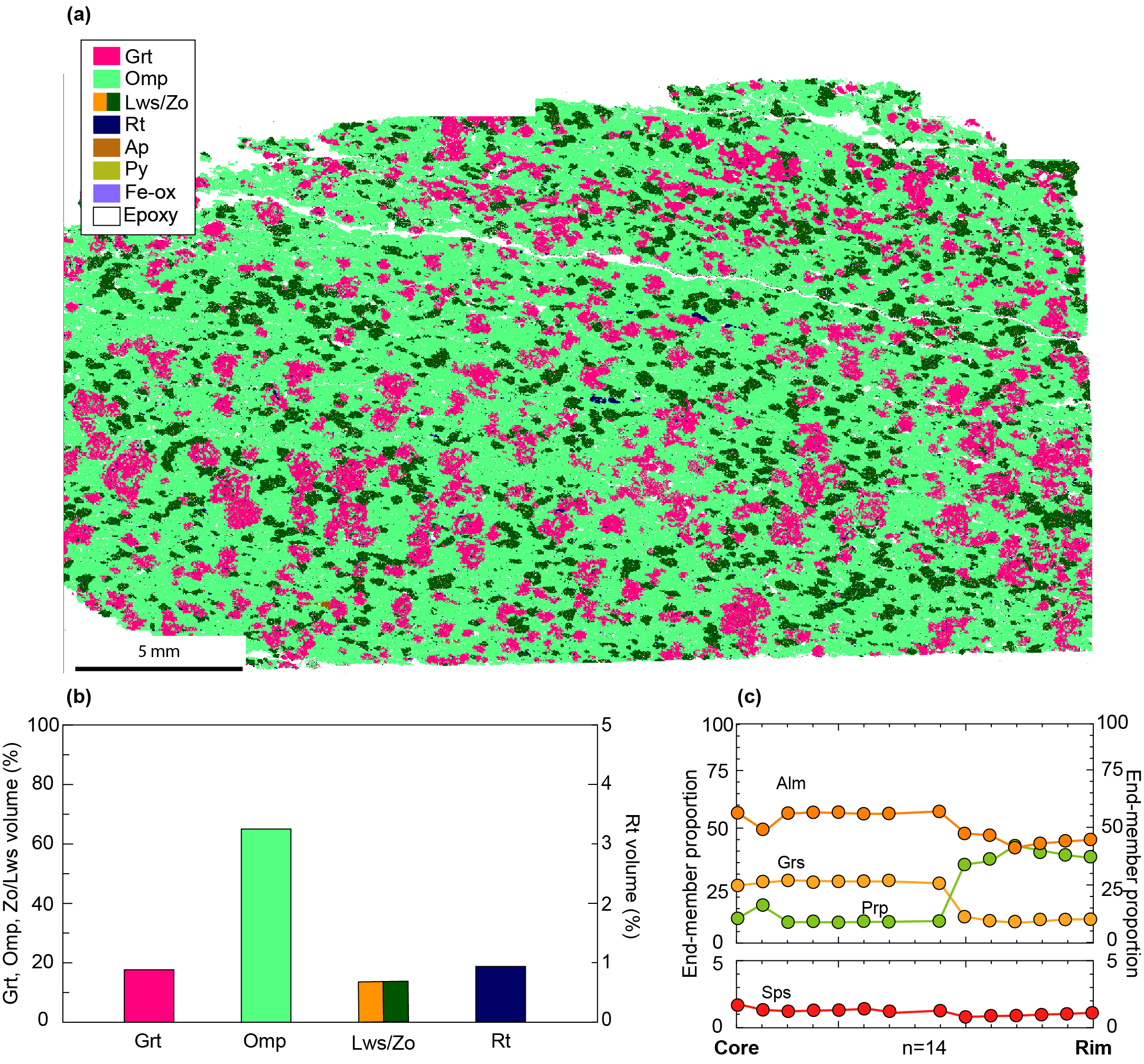
**

**Figure S4.** Petrology of eclogite xenolith 17MSR11. (a) Mineral identification and (b) volume proportions obtained from automated mineralogy. Grt = garnet, Omp = omphacite, Rt = rutile, Ap = apatite, Py = pyrite, Fe-ox = iron-oxides, Zo = zoisite, Lws = lawsonite. (c) Representative garnet analysis showing major element zoning in terms of end-member proportions: Alm = almandine [Fe^2+^/(Fe^2+^+Mg+Ca+Mn)], Prp = pyrope [Mg/(Fe^2+^+Mg+Ca+Mn)], Grs = grossular [Ca/(Fe^2+^+Mg+Ca+Mn)], Sps = spessartine [Mn/(Fe^2+^+Mg+Ca+Mn)].

**
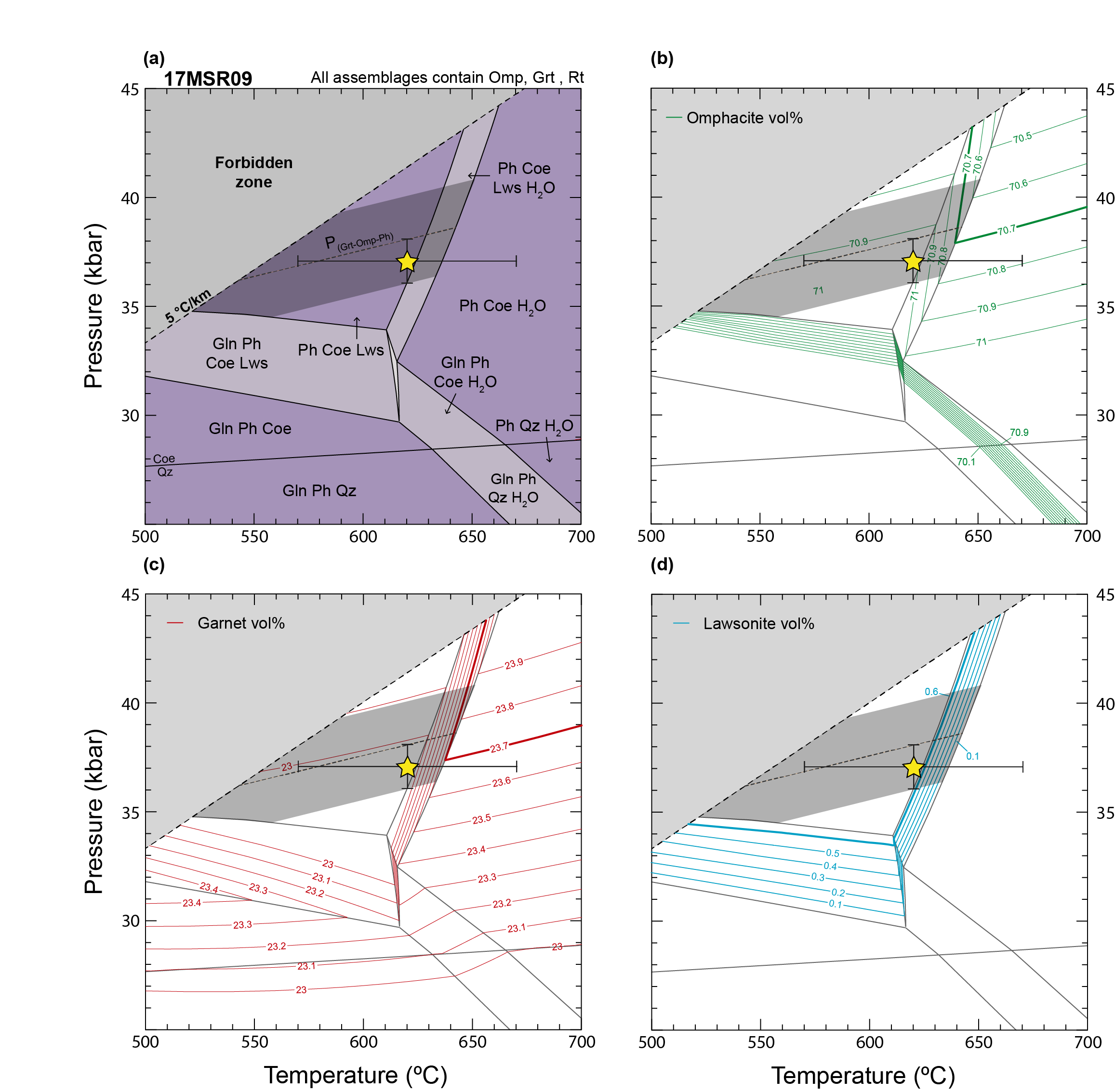
**

**Figure S5.** Petrological model for eclogite 17MSR09. (a) Pressure–temperature phase equilibrium diagram. (b–d) Isolines of equal volume proportion for (b) omphacite, (c) garnet, and (d) lawsonite. Solid thicker lines represent calculated mineral volume proportions matching observations. The yellow star indicates the peak *P–T* conditions that provide the best match between observed and calculated mineral proportions. The grey shaded area represents constraints from the garnet–omphacite–phengite barometry, with calculated conditions lying along the dashed line (P_Grt-Omp-Ph_) and an uncertainty envelope of ±2 kbar. Dashed error bars mark the extent of typical 2-sigma uncertainty in *P–T* estimation via this petrological modeling technique^8,9^. See methods for mineral abbreviations and details on the modeling.

**
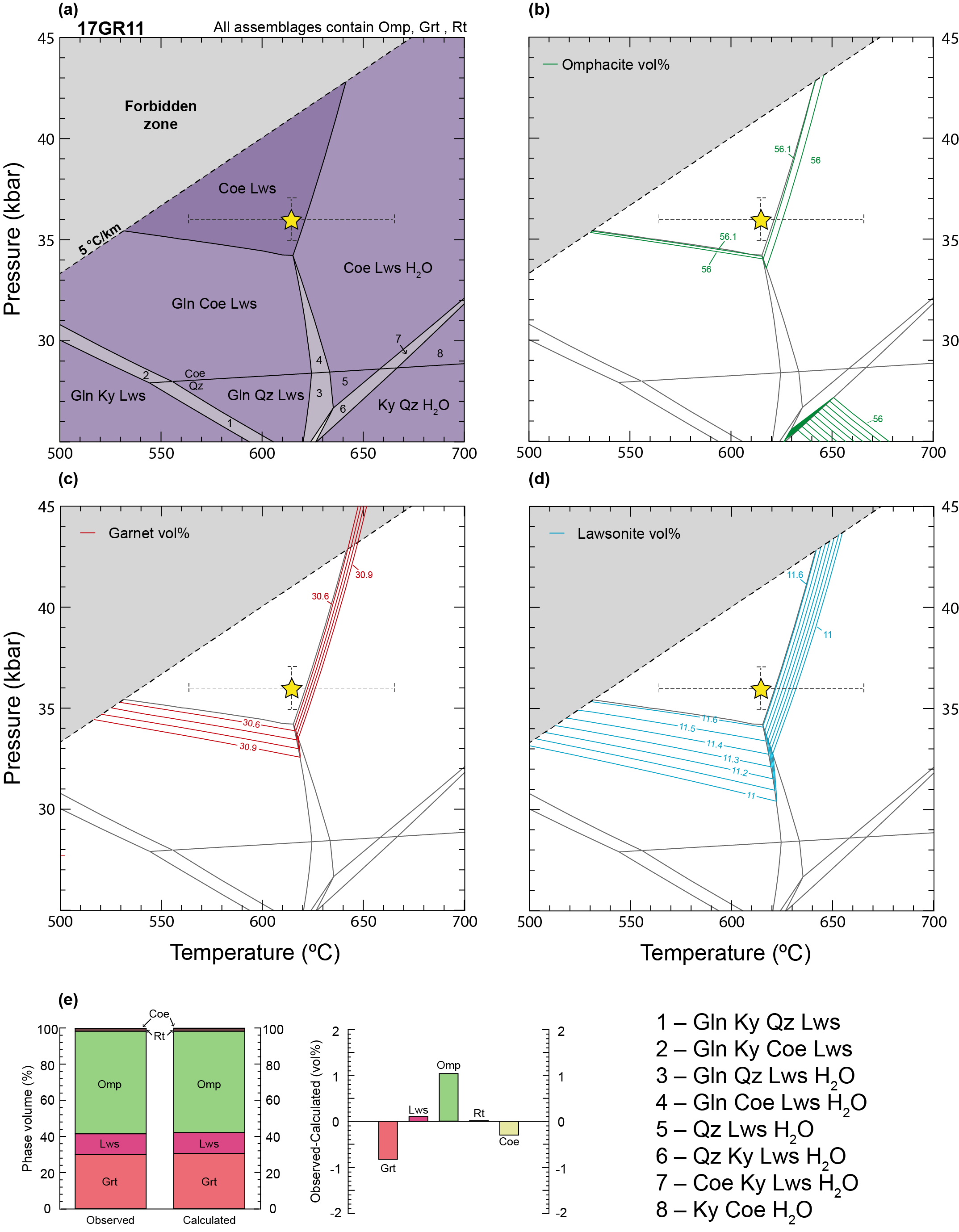
**

**Figure S6.** Petrological model for eclogite 17GR11. (a) Pressure–temperature phase equilibrium diagram. (b–d) Isolines of equal volume proportion for (b) omphacite, (c) garnet, and (d) lawsonite. (e) Comparison between the observed and calculated mineral proportions at ~36 kbar and ~625 °C, represented by the yellow star in (a). The yellow star indicates the peak *P–T* conditions that provide the best match between observed and calculated mineral proportions. Dashed error bars mark the extent of typical 2-sigma uncertainty in *P–T* estimation via this petrological modeling technique^8,9^. See methods for mineral abbreviations and details on the modeling.

**
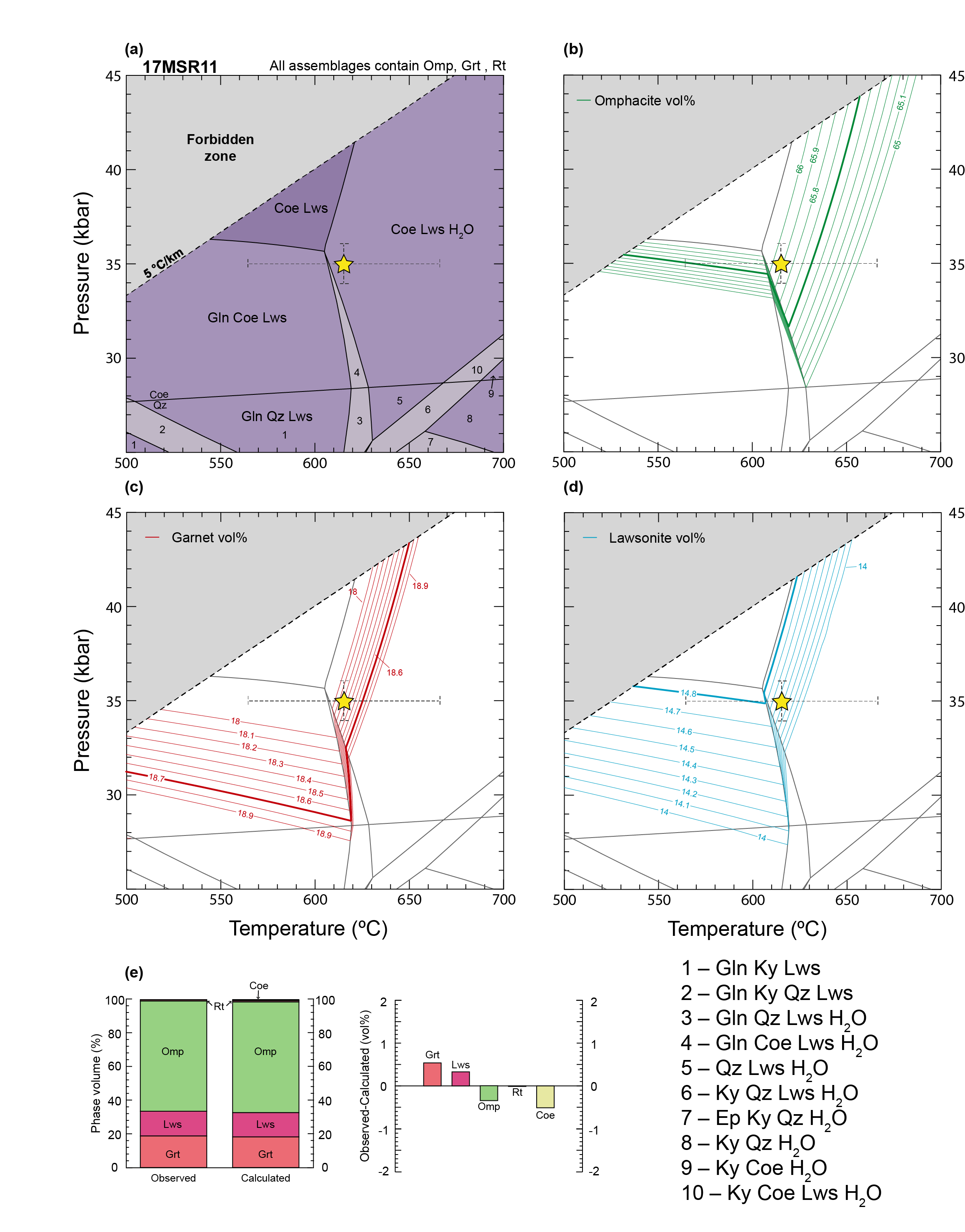
**

**Figure S7.** Petrological model for eclogite 17MSR11. (a) Pressure–temperature phase equilibrium diagram. (b–d) Isolines of equal volume proportion for (b) omphacite, (c) garnet, and (d) lawsonite. Solid thicker lines represent calculated mineral volume proportions matching observations. (e) Comparison between the observed and calculated mineral proportions at ~35 kbar and ~615 °C, represented by the yellow star in (a). The yellow star indicates the peak *P–T* conditions that provide the best match between observed and calculated mineral proportions. Dashed error bars mark the extent of typical 2-sigma uncertainty in *P–T* estimation via this petrological modeling technique^8,9^. See methods for mineral abbreviations and details on the modeling.

**Figure S8.** Uncertainty matrix showing the sensitivity of calculated slab dip angle according to distance from the trench and crustal thickness. The mean dip angle varies by ±0.6° for every 10 km uncertainty in proto-plateau continental crust thickness and ±0.3° for every 50 km variation in absolute distance from the trench.

**Figure S9.** Uncertainty matrix showing the sensitivity of calculated SCLM thickness according to different values of SLCM density and pressure difference between the continental Moho and the subducted slab top. The SCLM thickness varies by ±3 km for every 1 kbar of pressure variation and ±1 km for every 50 kg/m^3^ change in the density.

**SUPPLEMENTARY REFERENCES**

1. Hacker, B. R., Kelemen, P. B., & Behn, M. D. Differentiation of the continental crust by relamination. *Earth Planet. Sci. Lett.* **307**, 501–516 (2011).
2. Lee, C. T., Yin, Q., Rudnick, R. L., & Jacobsen, S. B. Preservation of ancient and fertile lithospheric mantle beneath the southwestern United States. *Nature*, **411**(6833), 69 (2001).
3. Liu, S., & Currie, C. A. Farallon plate dynamics prior to the Laramide orogeny: Numerical models of flat subduction. *Tectonophysics* **666**, 33–47 (2016).
4. Axen, G. J., van Wijk, J. W., & Currie, C. A. Basal continental mantle lithosphere displaced by flat-slab subduction. *Nat. Geosci.* **11**, 961–964 (2018).
5. Copeland, P., Currie, C. A., Lawton, T. F., & Murphy, M. A. Location, location, location: The variable lifespan of the Laramide orogeny. *Geology* **45**, 223–226 (2017).
6. Pearce, J. A., & J. R. Cann, Tectonic setting of basic volcanic rocks determined using trace element analyses. *Earth Planet. Sci. Lett.* **19**, 290–300 (1973).
7. Vermeesch, P. Tectonic discrimination diagrams revisited. *Geochem. Geophys. Geosyst.* **7**(6) (2006).
8. Powell, R., & Holland, T. J. B. On thermobarometry. *J. Metamorph. Geol.* **26**, 155–179 (2008).
9. Palin, R. M., Weller, O. M., Waters, D. J., & Dyck, B. Quantifying geological uncertainty in metamorphic phase equilibria modelling; a Monte Carlo assessment and implications for tectonic interpretations. *Geosci. Front.* **7**, 591–607 (2016).
